# A literature-based study of patient-centered care and communication in nurse-patient interactions: barriers, facilitators, and the way forward

**DOI:** 10.1186/s12912-021-00684-2

**Published:** 2021-09-03

**Authors:** Abukari Kwame, Pammla M. Petrucka

**Affiliations:** 1 College of Graduate and Postdoctoral Studies, University of Saskatchewan, Saskatoon, Canada; 2 College of Nursing, University of Saskatchewan, Regina Campus, Regina, Canada

**Keywords:** Patient-centered care, Therapeutic communication, Nurse-patient interactions, Clinical discourse space, Patient-centered care and communication continuum

## Abstract

Providing healthcare services that respect and meet patients’ and caregivers’ needs are essential in promoting positive care outcomes and perceptions of quality of care, thereby fulfilling a significant aspect of patient-centered care requirement. Effective communication between patients and healthcare providers is crucial for the provision of patient care and recovery. Hence, patient-centered communication is fundamental to ensuring optimal health outcomes, reflecting long-held nursing values that care must be individualized and responsive to patient health concerns, beliefs, and contextual variables. Achieving patient-centered care and communication in nurse-patient clinical interactions is complex as there are always institutional, communication, environmental, and personal/behavioural related barriers. To promote patient-centered care, healthcare professionals must identify these barriers and facitators of both patient-centered care and communication, given their interconnections in clinical interactions. A person-centered care and communication continuum (PC4 Model) is thus proposed to orient healthcare professionals to care practices, discourse contexts, and communication contents and forms that can enhance or impede the acheivement of patient-centered care in clinical practice.

## Background

Providing healthcare services that respect and meet patients’ and their caregivers’ needs are essential in promoting positive care outcomes and perceptions of quality of care, thus constituting patient-centered care. Care is “a feeling of concern for, or an interest in, a person or object which necessitates looking after them/it” [1]. The Institute of Medicine (IOM) noted that to provide patient-centered care means respecting and responding to individual patient’s care needs, preferences, and values in all clinical decisions [2]. In nursing care, patient-centered care or person-centered care must acknowledge patients’ experiences, stories, and knowledge and provide care that focuses on and respects patients’ values, preferences, and needs by engaging the patient more in the care process [3]. Healthcare providers and professionals are thus required to fully engage patients and their families in the care process in meaningful ways. The IOM, in its 2003 report on *Health Professions Education*, recognized the values of patient-centered care and emphasized that providing patient-centered care is the first core competency that health professionals’ education must focus on [4]. This emphasis underscored the value of delivering healthcare services according to patients’ needs and preferences.

Research has shown that effective communication between patients and healthcare providers is essential for the provision of patient care and recovery [5–8]. Madula et al. [6], in a study on maternal care in Malawi, noted that patients reported being happy when the nurses and midwives communicated well and treated them with warmth, empathy, and respect. However, other patients said poor communication by nurses and midwives, including verbal abuse, disrespect, or denial from asking questions, affected their perceptions of the services offered [6]. Similarly, Joolaee et al. [9] explored patients’ experiences of caring relationships in an Iranian hospital where they found that good communication between nurses and patients was regarded as “more significant than physical care” among patients.

According to Boykins [10], effective communication is a two-way dialogue between patients and care providers. In that dialogue, both parties speak and are listened to without interrupting; they ask questions for clarity, express their opinions, exchange information, and grasp entirely and understand what the others mean. Also, Henly [11] argued that effective communication is imperative in clinical interactions. He observed that health and illness affect the quality of life, thereby making health communication critical and that the “intimate and sometimes overwhelming nature of health concerns can make communicating with nurses and other healthcare providers very challenging” [11]. Furthermore, Henly [11] added that patient-centered communication is fundamental to ensuring optimal health outcomes, reflecting long-held nursing values that care must be individualized and responsive to patient health concerns. Given the prevalence of face-to-face and device-mediated communications and interactions in healthcare settings, we must explore and clarify who, what, where, when, why, and how interactions with individuals, families, and communities are receiving care and health services [11].

The value of effective communication in nurse-patient clinical interactions cannot be overemphasized, as “research has shown that communication processes are essential to more accurate patient reporting and disclosure” [12]. Respectful communication between nurses and patients can reduce uncertainty, enhance greater patient engagement in decision making, improve patient adherence to medication and treatment plans, increase social support, safety, and patient satisfaction in care [12, 13]. Thus, effective nurse-patient clinical communication is essential to enhancing patient-centered care and positive care outcomes.

Patient-centered communication, also known as person-centered communication or client-centered communication, is defined as a process that invites and encourages patients and their families to actively participate and negotiate in decision-making about their care needs, as cited in [7]. Patient-centered communication is crucial in promoting patient-centered care and requires that patients and their caregivers engage in the care process. As McLean [14] observed, patient-centered care can be enhanced through patient-centered communication by valuing patients’ dignity and rights. Through open communication and collaboration, where information and care plans are shared among care providers, patients, and their families, care provision becomes patient-centered [14].

Given the interconnected nature of patient-centered care and communication, we must identify the barriers and enablers of patient-centered care and communication and proposed efficient ways to enhance that because patient-centered communication is essential in achieving patient-centered care. Our aim in this paper is to identify the barriers and facilitators of patient-centered care and communication and propose and present a patient-centered care and communication continuum (PC4) Model to explain how patient-centered care can be enhanced in nurse-patient clinical interactions. As Grant and Booth argued, critical reviews are often used to present, analyse, and synthesized research evidence from diverse sources, the outcome of which is a hypothesis or a model as an interpretation of existing data to enhance evidence-based practice [15]. Thus, this critical literature review study explores the questions: what are the barriers and facilitators of patient-centered care and how can patient-centered care be enhanced through effective clinical communication?

An earlier version of this study was submitted as part of author AK’s doctoral comprehensive exams in February 2021. An interdisciplinary doctoral committee recommended many of the included literature and the questions explored in this study based on the current discourse of patient-centered care advocated for in many healthcare facilities and in recognition of the universal healthcare access objective of the health sustainable development goal. Additional searches for literature were conducted between September and November 2020 using keywords such as *barriers and facilitators of nurse-patient interaction, patient-centered care, patient-centered communication*, and *nurse-patient communication*. Databases searched included CINAHL, PubMed, Medline, and Google Scholar. Included studies in this critical review were empirical research on nurse-patient interactions in different care settings published in English and open access. All relevant articles were read, and their main findings relevant to our review questions were identified and organized into themes and subthemes discussed in this paper. Other published studies were read, and together with those that addressed the review question, a model was developed regarding how to enhance patient-centered care through effective communication.

## Main Text

### Barriers to Patient-Centered Care and Communication

Nurses constitute a significant workforce of care providers whose practices can severely impact care outcomes (both positive and negative). Nurses spend much time with patients and their caregivers. As a result, positive nurse-patient and caregiver relationships are therapeutic and constitute a core component of care [9, 13]. In many instances, nurses serve as translators or patients’ advocates, in addition to performing their primary care duties. Although good nurse-patient relationships positively impact nurse-patient communication and interaction, studies have shown that several factors impede these relationships with significant consequences on care outcomes and quality [6, 16, 17]. Thus, these barriers limit nurses’ and other care providers’ efforts to provide healthcare that meets patients’ and caregivers’ needs. We categorize the barriers to patient-centered care and communication into four kinds: institutional and healthcare system-related, communication-related, environment-related, and personal and behaviour-related barriers. Although these barriers are discussed in separate subheadings, they are interlinked in complex ways during clinical practice.

#### Institutional and Healthcare System Related Barriers

Many barriers to providing patient-centered care and communication during nurse-patient interactions emanate from healthcare institutional practices or the healthcare system itself. Some of these factors are implicated in healthcare policy or through management styles and strategies.

Shortage of nursing staff, high workload, burnout, and limited-time constituted one complex institutional and healthcare system-level barrier to effective care delivery [18, 19]. For instance, Loghmani et al. [20] found that staffing shortages prevented nurses from having adequate time with patients and their caregivers in an Iranian intensive care unit. Limitations in nursing staff, coupled with a high workload, led to fewer interactions between nurses, patients, and caregivers. Similarly, Anoosheh et al. [16] found that heavy nursing workload was ranked highest as a limiting factor to therapeutic communication in nurse-patient interactions in Iran.

In a study on communication barriers in two hospitals affiliated with Alborz University of Medical Sciences, Norouzinia et al. [21] found that shortage of nurses, work overload, and insufficient time to interact with patients were significant barriers to effective nurse-patient interactions. Similar factors are identified as barriers to nurse-patient communication and interactions in other studies [13, 16, 18]. For instance, Amoah et al. [16] reported that nursing staff shortage and high workload were barriers to patient-centered care and therapeutic communication among Ghanaian nurses and patients. Amoah and colleagues reported a patient’s statement that:

*[B]ecause there are few nurses at the ward, sometimes you would want a nurse to attend to you, but he or she might be working on another patient, so in such case, the nurse cannot divide him or herself into two to attend to you both* [16].

Nurses and patients and their caregivers have noted that limited time affects nurse-patient interactions, communication, and care quality. Besides, Yoo et al. [22] reported that limited visiting hours affected communications between caregivers and nurses in a tertiary hospital in Seoul, Korea. Since the caregivers had limited time to spend with patients, they had little knowledge about the intensive care unit and distrusted the nurses.

Although nursing staff shortage is a significant barrier to patient-centered care and communication that healthcare institutions and managers must know, some healthcare scholars have critique nurses’ complaints of time limitation. For instance, McCabe [7] argued that the quality of nurse-patient interactions is what matters and not the quantity of time spent with patients and their caregivers. McCabe maintained that “spending long periods with patients does not always result in positive nurse-patient relationships” [7]. He argued that implementing patient-centered care does not require additional time; hence, nurses’ perceptions of being too busy cannot excuse poor therapeutic communication during clinical interactions. Instead, nurses are encouraged to develop self-awareness, self-reflection, and a commitment to ensuring that patients receive the needed care.

Another institution-related barrier to patient-centered care and communication is the healthcare system’s emphasis on task-centered care. Care providers are more focused on completing care procedures than satisfying patients’ and caregivers’ needs and preferences. This barrier to patient-centered care and communication is acknowledged in several studies [7, 14, 20, 22, 23]. For example, McLean [14] studied dementia care in nursing homes in the United States. She found that patient-centered care and communication in one nursing home (Snow I) were severely affected when nurses, physicians, and care managers focused on completing tasks or observing care and institutional routines to the detriment of satisfying patients’ care needs. However, in the other care home (Snow II), patient-centered care was enhanced as nurses, physicians, and the care home managers focused on addressing patients’ needs and values rather than completing care routines and tasks.

Similarly, Yoo and colleagues [22] observed that nurse-patient communication was affected when the ICU nurses placed urgency on completing tasks linked directly to patients’ health (e.g., stabilizing vital signs) than communicating to addressed patients’ specific needs. This evidence shows that when nurses are more task-focused, patients and caregivers are treated as bodies and objects, on which medical and care practices must be performed to restore health. Research has shown that when nurses focus on task-oriented care, it becomes hard to provide holistic care to patients or teach and communicate with patients even when nurses are less busy [20].

Nursing managers and their management styles can affect patient-centered care and communication. Studies have revealed that the management styles that nursing managers implement can either facilitate or impede patient-centered care [14, 22]. When nurse managers orient their nursing staff towards task-centered care practices, it affects nurse-patient interaction and communication. Moreover, when nurse managers fail to address their staff’s mental health needs and personal challenges, it influences how nurses attend to patients’ care needs. For example, nurses have indicated that nurse-patient communication is affected when nurse managers are unsupportive or unresponsive to their needs [20].

In a study exploring nursing and midwifery managers’ perspectives on obstacles to compassion giving and therapeutic care across 17 countries, Papadopoulos et al. [24] discovered that nurses and midwifery managers’ characteristics and experiences could facilitate or impede compassion and therapeutic interactions in nursing care. Negative personal attitudes, including selfishness, arrogance, self-centeredness, rudeness, lack of leadership skills, the desire for power, and feelings of superiority among nurses and midwifery managers, were obstacles to compassion building. The study further showed that managers who emphasize rules, tasks, and results do not prioritize relationship-building and see their staff as workers rather than team members [24]. Therefore, nurse managers and care administrators must monitor nurse-patient interaction and communication to address nurses’ concerns and support them, especially in resource-constrained and high patient turnover contexts [25, 26].

#### Communication-Related Barriers

Effective communication is essential to providing patient-centered care. Studies have shown that poor communication between care providers and patients and their caregivers affects care outcomes and perceptions of care quality [7, 16, 27, 28]. A consistent communication-related barrier in nurse-patient interaction is miscommunication, which often leads to misunderstandings between nurses, patients, and their families [20]. Other communication-related barriers include language differences between patients and healthcare providers [6, 16, 27], poor communication skills, and patients’ inability to communicate due to their health state, especially in ICU, dementia, or end-of-life care contexts [13, 22]. For instance, in their maternity care study, Madula et al. [6] noted that language barriers significantly affected effective communication between nurses/midwives and expectant mothers. A patient in their study indicated that although many nurses were polite and communicated well, some nurses had challenges communicating with patients in the Chitumbuka language, which affected those nurses’ ability to interact effectively with patients [6].

Furthermore, Norouzinia et al. [21] asserted that effective communication could not be established when nurses and patients have a language difference. Moreover, the meanings of certain non-verbal communication acts (e.g., head nodding, eye gaze, touch) can invoke different interpretations across different cultures, which could impede the interactions between patients and nurses. Even in healthcare contexts where nurses and patients speak the same language, “differences in vocabulary, rate of speaking, age, background, familiarity with medical technology, education, physical capability, and experience can create a huge cultural and communication chasm” between nurses and patients [12]. In ICU and other similar care settings, nurses find it difficult to effectively communicate with patients because the mechanical ventilators made it hard for patients to talk [22].

To overcome the communication-related barriers, healthcare institutions must make it a responsibility to engage translators and interpreters to facilitate nurse-patient interactions where a language barrier exists. Moreover, nurses working in ICU and other similar settings should learn and employ alternative forms of communication to interact with patients.

#### Environment-Related Barriers

The environment of the care setting can impact nurse-patient communication and the resulting care. Thus, “good health care experiences start with a welcoming environment” [29]. Mastors believed that even though good medicine and the hands working to provide care and healing to the sick and wounded are essential, we must not “forget the small things: a warm smile, an ice chip, a warm blanket, a cool washcloth. A pillow flipped to the other side and a boost in bed” [29]. The environment-related barriers are obstacles within the care setting that inhibit nurse-patient interaction and communication and may include a noisy surrounding, unkept wards, and beds, difficulties in locating places, and navigating care services. Noisy surroundings, lack of privacy, improper ventilation, heating, cooling, and lighting in specific healthcare units can affect nurse-patient communication. These can prevent patients from genuinely expressing their healthcare needs to nurses, which can subsequently affect patient disclosure or make nursing diagnoses less accurate [13, 18, 21]. For instance, Amoah et al. [16] revealed that an unconducive care environment, including noisy surroundings and poor ward conditions, affected patients’ psychological states, impeding nurse-patient relationships and communication. Moreover, when care services are not well-coordinated, new patients and their caregivers find it hard to navigate the care system (e.g., locating offices for medical tests and consultations), which can constrain patient-centered care and communication.

Reducing the environment-related barriers will require making the care setting tidy/clean, less noisy, and coordinating care services in ways that make it easy for patients and caregivers to access. Coordinating and integrating care services, making care services accessible, and promoting physical comfort are crucial in promoting patient-centered care, according to *Picker’s Eight Principles of Patient-Centered Care* [30].

#### Personal and Behaviour Related Barriers

The kind of nurse-patient relationships established between nurses and patients and their caregivers will affect how they communicate. Since nurses and patients may have different demographic characteristics, cultural and linguistic backgrounds, beliefs, and worldviews about health and illnesses, nurses’, patients’, and caregivers’ attitudes can affect nurse-patient communication and care outcomes. For instance, differences in nurses’ and patients’ cultural backgrounds and belief systems have been identified as barriers to therapeutic communication and care [12, 13, 21]. Research shows that patients’ beliefs and cultural backgrounds affected their communication with nurses in Ghana [16]. These scholars found that some patients refused a blood transfusion, and Muslim patients refused female nurses to attend to them because of their religious beliefs [16]. Further, when nurses, patients, or their caregivers have misconceptions about one another due to past experiences, dissatisfaction about the care provided, or patients’ relatives and caregivers unduly interfere in the care process, nurse-patient communication and patient-centered care were affected [16, 21].

Similarly, nurse-patient communication was affected when patients or caregivers failed to observe nurses’ recommendations or abuse nurses due to misunderstanding [20], while patients’ bad attitudes or disrespectful behaviours towards nurses can inhibit nurses’ ability to provide person-centered care [31]. The above-reviewed studies provided evidence on how patients’ and caregivers’ behaviours can affect nurses’ ability to communicate and deliver patient-centered care.

On the other hand, nurses’ behaviours can also profoundly affect communication and care outcomes in the nurse-patient dyad. When nurses disrespect, verbally abuse (e.g., shouting at or scolding), and discriminate against patients based on their social status, it affects nurse-patient communication, care outcomes, and patient disclosure [6, 32]. For instance, Al-Kalaldeh et al. [18] believe that nurse-patient communication is challenged when nurses become reluctant to hear patients’ feelings and expressions of anxiety. When nurses ignore patients’ rights to share ideas and participate in their care planning, such denials may induce stress, discomfort, lack of trust in nurses, thereby leading to less satisfaction of care [18].

Furthermore, when nurses fail to listen to patients’ and caregivers’ concerns, coerce patients to obey their rules and instructions [16, 17, 20], or fail to provide patients with the needed information, nurse-patient communication and patient-centered care practices suffer. To illustrate, in Ddumba-Nyanzia et al.‘s study on communication between HIV care providers and patients, a patient remarked that: “I realized no matter how much I talked to the counselor, she was not listening. She was only hearing her point of view and nothing else, [and] I was very upset” [17]. This quote indicates how care provider attitudes can constrain care outcomes. Due to high workload, limited time, poor remunerations, and shortage of personnel, some nurses can develop feelings of despair, emotional detachment, and apathy towards their job, which can lead to low self-esteem or poor self-image, with negative consequences on nurse-patient interactions [13, 18].

Given the significance of effective communication on care, overcoming the above personal and behaviour related barriers to patient-centered care and communication is crucial. Nurses, patients, and caregivers need to reflect on the consequences of their behaviours on the care process. Thus, overcoming these barriers begins with embracing the facilitators of patient-centered care and communication, which we turn to in the next section.

### Facilitators of patient-centered care and communication

Patient-centered care and communication can be facilitated in several ways, including building solid nurse-patient relationships.

First, an essential facilitator of patient-centered care and communication is overcoming practical communication barriers in the nurse-patient dyad. Given the importance of communication in healthcare delivery, nurses, patients, caregivers, nursing managers, and healthcare administrators need to ensure that effective therapeutic communication is realized in the care process and becomes part of the care itself. Studies have shown that active listening among care providers is essential to addressing many barriers to patient-centered care and communication [7, 13]. Although handling medical tasks promptly in the care process is crucial, the power of active listening is critical, meaningful, and therapeutic [22]. By listening to patients’ concerns, nurses can identify patients’ care needs and preferences and address their fears and frustrations.

Another facilitator of patient-centered care is by understanding patients and their unique needs [25], showing empathy and attending attitudes [7, 13], expressing warmth and respect [22], and treating patients and caregivers with dignity and compassion as humans. For instance, McCabe [7] noted that attending, which obligates nurses to demonstrate that they are accessible and ready to listen to patients, is a patient-centered care process; a fundamental requirement for nurses to show genuineness and empathy, despite the high workload. Showing empathy, active listening, respect, and treating patients with dignity are core to nursing and care, and recognized in the *Code of Ethics for Nurses* [33], and further emphasized in the ongoing revision of the *Code of Ethics for nurses* [34].

Besides, engaging patients and caregivers in the care process through sharing information, inviting their opinion, and collaborating with them constitutes another facilitator of patient-centered care and communication. When patients and caregivers are engaged in the care process, misunderstandings and misconceptions are minimized. When information is shared, patients and caregivers learn more about their health conditions and the care needed. As McLean [14] argued, ensuring open communication between care providers and patients and their families is essential to enhancing patient-centered care. Conflicts ensue when patients or their families are denied information or involvement in the care process. As a result, the Harvard Medical School [30] identified patient engagement, information sharing, and nurse-patient collaboration during care as essential patient-centered care principles.

Finally, health policy must be oriented towards healthcare practices and management to facilitate patient-centered care and communication. These policies, at a minimum, can involve changes in management styles within healthcare institutions, where nurse managers and healthcare administrators reflect on nursing and care practices to ensure that the *Code of Ethics of Nurses* and patients’ rights are fully implemented. Resource constraints, staff shortages, and ethical dilemmas mainly affect care practices and decision-making. Nonetheless, if patients are placed at the center of care and treated with dignity and respect, most of the challenges and barriers of patient-centered care will diminish. Empowering practicing nurses, equipping them with interpersonal communication skills through regular in-service training, supporting them to overcome their emotional challenges, and setting boundaries during nurse-patient interactions will enhance patient-centered care practices.

In line with the above discussion, Camara et al. [25] identify three core dimensions that nurses, patients, and caregivers must observe to enhance patient-centered care: treating the patient as a person and seeing the care provider as a person and a confidant. Regarding the first dimension, care providers must welcome patients, listen to them, share information with them, seek their consent, and show them respect when providing care. The second dimension requires that the healthcare provider be seen and respected as a person, and negative perceptions about care providers must be demystified. According to Camara et al. [25], care providers must not overemphasize their identities as experts but rather establish good relationships with patients to understand patients’ personal needs and problems. Lastly, patients and caregivers must regard care providers as confidants who build and maintain patients’ trust and encourage patients’ participation in care conversations. With this dimension, patients and caregivers must know that nurses and other care providers have the patient at heart and work to meet their care needs and recovery process.

Camara et al.‘s [25] three dimensions are essential and position patients, their caregivers, and nurses as partners who must engage in dialogic communication to promote patient-centered care. As a result, effective communication, education, and increased health literacy among patients and caregivers will be crucial in that direction.

### Enhancing Patient-Centered Care and Communication: A Proposed Model

Nursing care practices that promote patient-centered communication will directly enhance patient-centered care, as patients and their caregivers will actively engage in the care process. To enhance patient-centered communication, we propose person-centered care and communication continuum (PC4) as a guiding model to understand patient-centered communication, its pathways, and what communication and care practices healthcare professionals must implement to achieve person-centered care. In this PC4 Model, we emphasize the person instead of the patient because they are a person before becoming a patient. Moreover, the PC4 Model is supposed to apply to all persons associated with patient care; thus, respect for the dignity of their personhood is crucial.

Although much is written about patient-centered communication in the healthcare literature, there is a gap regarding its trajectory and what communication content enhances patient-centered communication. Also, little is known about how different clinical discourse spaces influence communication and its content during nurse-patient clinical interactions. Using evidence from Johnsson et al. [3], Murira et al. [23], and Liu et al. [35], among other studies, we outline the components of the PC4 Model and how different discourse spaces in the clinical setting and the content of communication impact patient-centered care and communication.

**Fig. 1:**
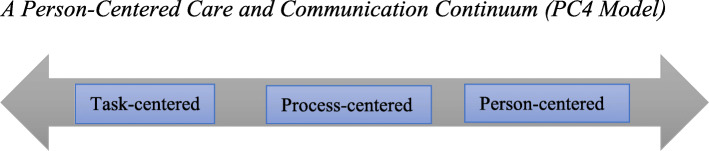
A Person-Centered Care and Communication Continuum (PC4 Model)

The proposed PC4 Model in this paper has three unbounded components based on the purpose of and how communication is performed among care providers, patients, and their caregivers. Figure 1 illustrates the PC4 Model, its features, and trajectory.

#### Task-Centered Communication

At the lowest end of the PC4 Model is task-centered communication. Here, the care provider’s role is to complete medical tasks as fast as possible with little or no communication with the patient and their caregivers. Patients and caregivers are treated as bodies or objects whose disease symptoms need to be studied, identified, recorded, treated, or cured. As Johnsson et al. [3] observed, communication content at this stage is mainly biomedically oriented, where nurses and other healthcare professionals focus on the precise medical information (e.g., history taking, medical examination, test results, medication, etc.) about the patient. With a task-centered orientation, nurses make journal entries about their patients’ disease state and ensure that treatment plans, diagnostic tests, and medical prescriptions are completed. Communication at this stage is often impersonal or rigid (see [23] for details). Care providers may address patients and their caregivers by using informal attributes (e.g., bed 12, the woman in the red shirt, card 8, etc.), thereby ignoring patients’ and caregivers’ personal and unique identities. Patients’ and caregivers’ nonverbal communication signs are mostly overlooked.

Motivations for task-centered communication can be attributed to time limitation, high workload, and staff shortage, thereby pushing nurses and other care providers to reach as many patients as possible. Moreover, the healthcare system’s orientation towards and preference for biomedically-focused care seems to favour task-centered communication [7, 14].

Depending on the clinical discourse space under which patient-provider interactions occur, different forms of communication are employed. Clinical discourse spaces can be public (e.g., in the ward, patient bedside), private (e.g., consulting rooms, medical test labs, nurse staff station, etc.), or semi-private (e.g., along the corridor) [35]. In these clinical discourse spaces, nurse-patient communication can be uninformed (patients or caregivers are not informed about patients’ care conditions or why specific data and routines are performed). It can be non-private (others can hear what the nurse and patient are talking about) or authoritative (care providers demonstrate power and control and position themselves as experts) [23]. Finally, in task-centered communication, healthcare providers often use medical jargon or terminologies [3] since the goal of communication is not to engage the patient in the process. Usually, patients or their caregivers are not allowed to ask questions, or their questions get ignored or receive superficial, incomprehensible responses.

#### Process-Centered Communication

Process-centered communication is an intermediate stage on the continuum, which could slip back into the task-centered or leap forward into person-centered communication. Through process-centered communication, care providers make an effort to know patients and their caregivers as they perform care routines. Care providers ask patients or their caregivers questions to understand the care conditions but may not encourage patients or caregivers to express their thoughts about their care needs. Patients and caregivers are recognized as persons with uniques care needs but may not have the agency to influence the care process. Care providers may chit-chat with patients or their caregivers to pass the time as they record patients’ medical records or provide care. Unlike task-centered communication, there is informative and less authoritative communication between nurses and patients and their caregivers. The goal of process-centered communication could be a mixture of instrumental and relational, with less display of power and control by nurses.

#### Person-Centered Communication

This is the highest point of the PC4 Model, where patient-centered care is actualized. At this stage of the communication continuum, patients and caregivers are treated as unique persons with specific care needs and are seen as collaborators in the care process. As McLean [14] observed, caregiving becomes a transactional relationship between the care provider and receiver at the person-centered stage of the continuum. The care itself becomes intersubjective, a mutual relational practice, and an ongoing negotiation for care providers and receivers [14].

The content of communication at this stage of the continuum is both “personal” and “explanatory” [3]. Nurses and other healthcare providers create meaningful relationships with patients and their caregivers, understand patients’ concerns, needs, and problems, use open-ended questions to encourage patients or caregivers to express their thoughts and feelings about the care situation. Nurses and other healthcare professionals explain care routines, patients’ health conditions, and management plans in lay language to patients and caregivers through person-centered communication. Accomplishing this level includes employing alternative forms of communication to meet the needs of intensive care unit patients, deaf patients, and ventilated and intubated patients. For instance, it has been shown that “deaf people […] frequently do not have access to clear and efficient communication in the healthcare system, which deprives them of critical health information and qualified health care” [36]. Empathetic communication practices, including active listening, showing genuine interest in patients’ care, and respect and warmth, become a significant part of nursing care [3, 7, 14, 22].

Different communication strategies are employed based on the care situation and context. Chit-chatting, as a form of personal communication [3], use of humor as a communication strategy [7, 8], and even maintaining silence [28] are essential in enhancing person-centered care and communication. Both care providers and patients or their caregivers use relationship-building and -protecting humor (see [28] for details) to address difficult situations in the care process.

### Implications of the PC4 Model for Nursing Practice

Given the values of effective communication in nurse-patient interactions and care outcomes, nurses and other healthcare providers must ensure that they develop therapeutic relationships with patients, their families, and caregivers to promote person-centered care and communication. Achieving that begins with knowing and reflecting on the barriers of therapeutic communication and ways to minimize them. The PC4 Model draws nurses and all healthcare providers’ attention to patient-centered care pathways and how effective communication is necessary. Healthcare professionals, including nurses, must be aware of how their communication orientation–––either oriented toward completing tasks, following care processes or toward addressing patients’ and their caregivers’ needs––can impact patient-centered care. Healthcare providers must observe the care context, patients’ unique situations, their non-verbal language and action, and whether they belong to historically marginalized groups or cultures.

Mastors [29] has offered healthcare providers some guidance to reflect on as they communicate and interact with patients and caregivers. Thus, (a) instead of asking patients, “What’s the matter?“ care providers must consider asking them, “What’s important to you?“ With this question, the patient is given a voice and empowered to contribute to their own care needs. Care providers should (b) check with patients in the waiting room to update patients whose waiting time has been longer than usual, based on the care context. They should also (c) try to remember their conversations with patients to build on them during subsequent interactions. This continuity can be enhanced by nurse managers reexamining how they deploy care providers to patients. The same nurse can be assigned to the same patients for the duration of the patient’s stay to help patients feel valued and visible [29].

Knowledge of cultural competence, sensitivity, humility, and interpersonal communication skills will help achieve and implement the PC4 Model. As Cuellar [37] argues, “[h]umility is about understanding and caring for all people [and] being empathetic.“ Cultural competence is a “dynamic process of acquiring the ability to provide effective, safe, and quality care to the patients through considering their different cultural aspects” [38]. The concept of cultural competence entails “cultural openness, awareness, desire, knowledge and sensitivity” during care [39]. It demands that care providers respect and tailor care to align with patients’ and caregivers’ values, needs, practices, and expectations, based on care and moral ethics and understanding [39]. Active listening and showing compassion as therapeutic relationship-building skills are essential, and continuous education and mentorship will be crucial to developing these skills among healthcare providers.

We invite qualitative and quantitative studies, especially on language use and communication strategies, to explore and evaluate the PC4 Model. Providing in-depth and experiential data on ways to increase its effectiveness as a tool to guide healthcare providers is highly desired. More knowledge can support healthcare providers in offering evidence-based patient-centered care in different healthcare settings and units.

## Conclusions

Effective communication is an essential factor in nurse-patient interactions and a core component of nursing care. When communication in the nurse-patient dyad is patient-centered, it becomes therapeutic. It allows for trust and mutual respect in the care process, thereby promoting care practices that address patients’ and caregivers’ needs, concerns, and preferences. We have identified the barriers and facilitators of patient-centered care and communication and proposed a person-centered care and communication continuum (PC4 Model) to demonstrate how patient-centered communication intersects with patient-centered care.

## Data Availability

Not applicable.

## Abbreviations

ICU: Intensive Care Unit
IOM: Institution of Medicine
PC4: Person-Centered Care and Communication Continuum
